# Characterizing the influence of transportation infrastructure on Emergency Medical Services (EMS) in urban area—A case study of Seoul, South Korea

**DOI:** 10.1371/journal.pone.0183241

**Published:** 2017-08-14

**Authors:** Jungwoo Cho, Myoungsoon You, Yoonjin Yoon

**Affiliations:** 1 Department of Civil and Environmental Engineering, Korea Advanced Institute of Science and Technology (KAIST), Daejeon, South Korea; 2 Department of Public Health Science, Graduate School of Public Health, and Institute of Health and Environment, Seoul National University, Seoul, South Korea; Boston University, UNITED STATES

## Abstract

In highly urbanized area where traffic condition fluctuates constantly, transportation infrastructure is one of the major contributing factors to Emergency Medical Service (EMS) availability and patient outcome. In this paper, we assess the impact of traffic fluctuation to the EMS first response availability in urban area, by evaluating the k-minute coverage under 21 traffic scenarios. The set of traffic scenarios represents the time-of-day and day-of-week effects, and is generated by combining road link speed information from multiple historical speed databases. In addition to the k-minute area coverage calculation, the k-minute population coverage is also evaluated for every 100m by 100m grid that partitions the case study area of Seoul, South Korea. In the baseline case of traveling at the speed limit, both the area and population coverage reached nearly 100% when compared to the five-minute travel time national target. Employing the proposed LoST (Loss of Serviceability due to Traffic) index, which measures coverage reduction in percentage compared to the baseline case, we find that the citywide average LoST for area and population coverage are similar at 34.2% and 33.8%. However, district-wise analysis reveals that such reduction varies significantly by district, and the magnitude of area and population coverage reduction is not always proportional. We conclude that the effect of traffic variation is significant to successful urban EMS first response performance, and regional variation is evident among local districts. Complexity in the urban environment requires a more adaptive approach in public health resource management and EMS performance target determination.

## Introduction

Emergency Medical Service (EMS) is a time-critical service that depends on other critical infrastructures including transportation network. In highly urbanized area where traffic condition fluctuates constantly, transportation infrastructure is one of the major contributing factors to reliable EMS serviceability and patient outcome [1–6]. Understanding dependency of EMS on transportation infrastructure requires a multi-faceted analysis incorporating diverse information such as EMS dispatch locations, transportation network topology, and traffic speed information.

One of the most common EMS serviceability performance measures is the k-minute coverage area, which calculates the maximum area that can be reached within k-minute from a specific EMS dispatch location. Although there have been numerous researches to evaluate the k-minute EMS coverage on transportation network based on Geographic Information System (GIS) [7–12], one fundamental shortcoming of most GIS-based studies is that EMS coverage area is calculated with a simple set of assumptions on travel distance and speed, such as Euclidean distance and speed limits. Although such assumptions on travel distance and speed are often accepted, they might not reflect the reality in a highly urbanized environment, where temporal and geographical traffic fluctuations are too diverse to model with simple preset values. Although recent adoption of real-time speed information bridges some of the gaps between simple preset speed values and reality [13,14], it only applies to the tactical decision of the current event, lacking the system-wide strategic EMS policy perspectives.

In this research, we generate 21 traffic scenarios, representing speed variation in three times of day and seven days of week time periods, based on multiple historical speed datasets. More specifically, 21 *speed profiles* are generated based on multiple citywide speed data sources for each link in the road network topology. Once the speed profiles are imposed on GIS, shortest-time travel routes from the EMS dispatch location are calculated. As a baseline case, traveling at speed limit is evaluated and used in the performance analysis.

The performance of EMS serviceability is measured in coverage reduction in percentage compared to the baseline case of speed limit, which we call the Loss of Serviceability due to Traffic (LoST). In addition to the commonly adopted k-minute coverage area, we also evaluate the *k-minute population coverage* to assess the influence of traffic variation to the population served. A case study is conducted in the city of Seoul with travel time target of 5 minute using total of 7,035 speed profiles for 335 road links. GIS analysis is performed using 100m by 100m grids to achieve effective and flexible aggregation for further statistical analyses.

### Current literatures

There have been numerous efforts to measure the EMS coverage, ranging from simple assumption of Euclidean distance to sophisticated method of network-based service area calculation. For example, Bauer et al. measured the area coverage by generating a circle with a radius of 10 km around the health care facility [15]. Others have adopted a more refined approach considering the transportation network topology. Liu et al. and Tansley et al. generated network-based area coverage within network distances of fixed kilometers to identify regions with low accessibility [8,11]. Peleg et al. produced 8-minute service areas to assess the performance of EMS response by counting the number of past incidents located in the service areas [7]. GIS is the preferred analysis tool implemented in a number of similar studies because of its advantages in a) storing road networks and origin/destination information in databases, b) calculating costs between origins and destinations on transportation networks with traffic delays, and c) presenting results under a certain time or distance threshold [16].

Despite the efforts to integrate transportation networks in the assessment of EMS serviceability, most studies assumed that ambulances travel at road speed limits [8,10,12,17]. Such assumption has been widely adopted in several EMS ambulance travel time prediction models [18–21], as well as in various ambulance allocation and reallocation models [9,22].

There also have been several attempts to utilize the historical speed data; Peleg and Pliskin utilized district-level historical traffic data to assign ambulance speeds that vary by district and by time [7]. More recently, Lam et al. used EMS log data to model travel time in congested and uncongested hours [9]. Although those studies succeeded in overcoming the simple assumption on travel speed, the extent of utilized speed information is limited to district, ignoring the link-level variation in the road network. Adopting speed values at the road link-level further improves the robustness of traffic modeling such as shortest-time travel route estimation, especially in highly urbanized areas. However, challenges lie in (1) whether such data is obtainable, and (2) how one should measure the EMS travel time performance with such dataset. In this paper, we address both challenges by conducting a case study of Seoul, South Korea, where link-level speed dataset is available, and travel time performance is measured by EMS coverage reduction both in geographical area and population.

## Materials and methods

**Fig 1:**
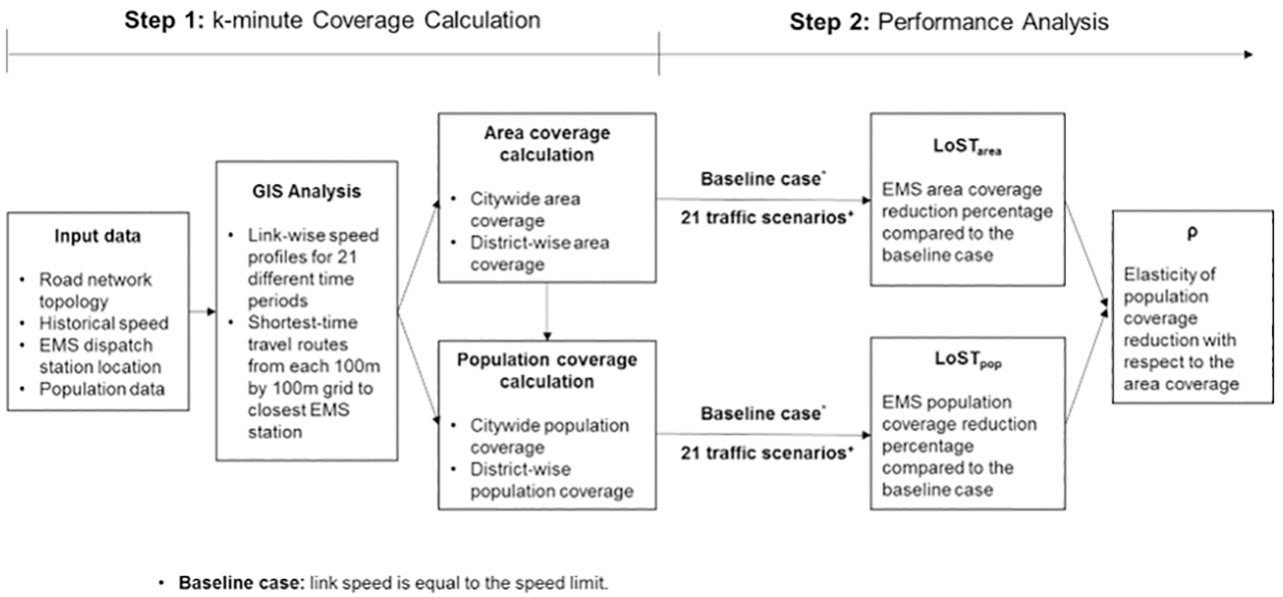
Two-step modeling and analysis framework to assess the impact of transportation infrastructure to EMS.

Analysis is carried out in two steps as illustrated in Fig 1. In the first step, the 21 link-wise speed profiles are generated using historical speed data, which represents speed variation in three times of day and seven days of week periods. Speed profiles are imposed on the road network topology using GIS, in which the study area is partitioned into 100m by 100m grids to allow flexibility for further analyses. Both geographical area and population coverage are calculated based on the shortest-time routes on the actual road network, and coverage is summarized at the citywide and district-wise level. In the following step, performance analysis is conducted based on the proposed Loss of Serviceability due to Traffic (LoST) index, and the elasticity between the area and population reduction is measured.

### Study area

**Fig 2:**
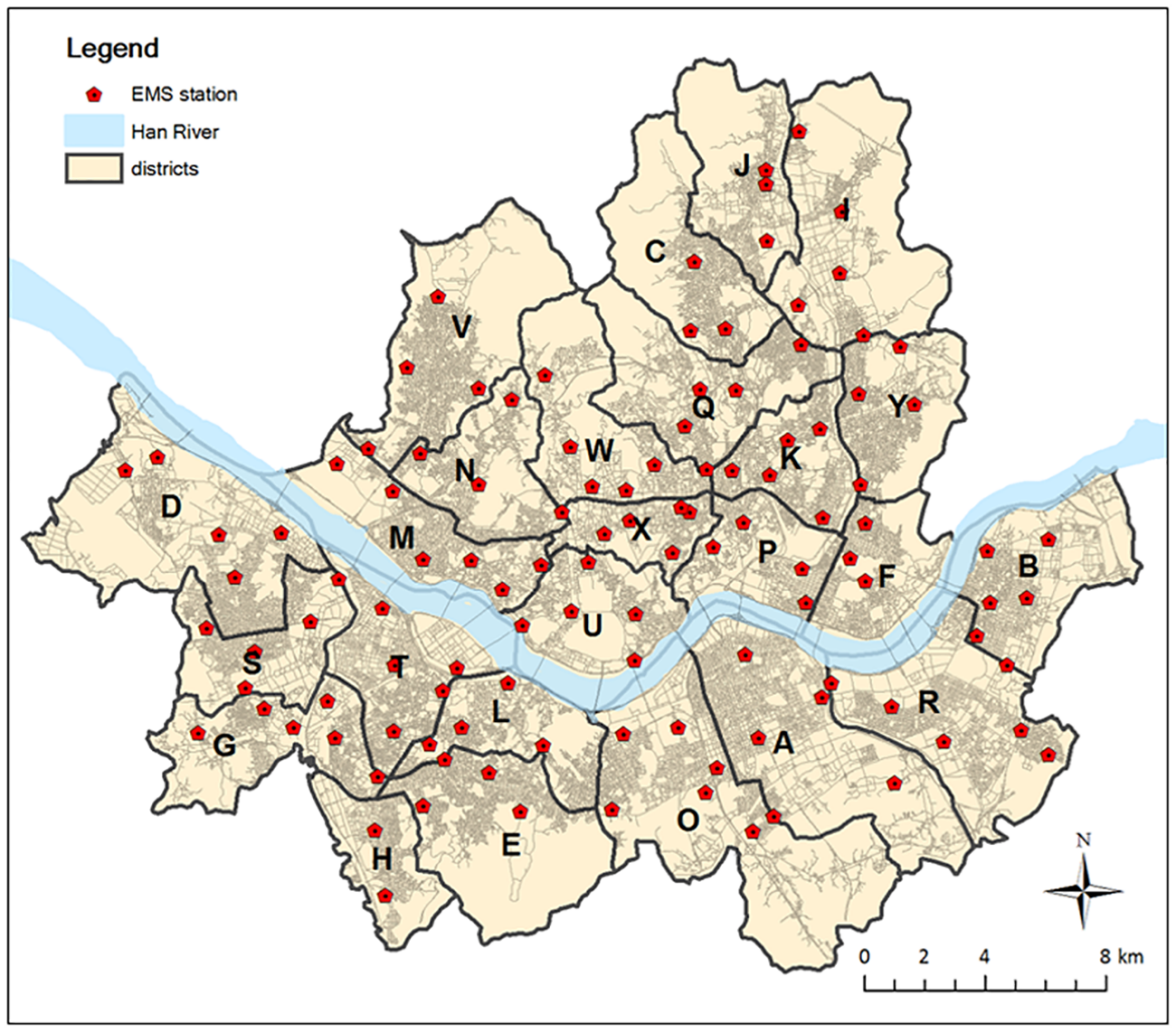
25 local districts and 114 EMS dispatch station locations in Seoul, S. Korea.

We select the city of Seoul, S. Korea as our case study area. The city is populated with more than 10 million residents, which is more than one fifth of the nation’s total population. The city is divided into 25 local districts, and each individual district is served by 3 to 8 EMS stations as shown in Fig 2. There are 114 EMS stations in total, among which 103 are equipped with a single ambulance, while the remaining 11 stations are equipped with two ambulances. A typical EMS response vehicle is staffed by three personnel, one EMT-basic, one EMT-intermediate, and one trained driver [23]. The city enacted a five-minute golden time rule in 2014, which aims to achieve 74% EMS calls responded within 5 minutes by 2017 [24].

### Data

#### EMS dispatch station location

Location of 114 ambulance stations in Seoul is collected from the Korea Transport Database, and each station is geo-located on ArcGIS 10.1.

#### Transportation network topology

Road network topology of Seoul is extracted from Korea Transport Database, and ArcGIS Network Analyst tool is used to analyze the road network.

#### Population data

Population data is obtained from XsDB of BIZGIS, which is available in 100m by 100m grid unit. Population data which totals for 29,111 grid squares were collected.

#### Road link speed

We employed two publicly available speed data sources to generate road link speed profiles for 21 traffic scenarios—the link-level annual average speed database and the district-wise annual average speed database. The link-level database contains annual average speed of 335 road links for three time-of-day categories: morning peak (07:00–10:00), afternoon off-peak (12:00–15:00), and evening peak (17:00–20:00). On the other hand, the district-wise database provides the annual average speed of the district for each of seven days of week. Combining two speed data sources yields 21 speed profiles for each road link, as explained in the following subsection.

### Link speed profile generation

Since link-level speed is provided only by time of day without the day-of-week information, the district-wise day-of-week speed information is utilized to obtain the weight factor $\frac{v_{n,d}}{\bar{v_{n,d}}}$, which is then applied to the time-of-day speed value to generate the link speed by day of week. Specifically, for each link *l* in the road network, the speed at time period *p* on day *d* is obtained as shown below.

$$v_{l}^{s}=v_{l}^{p}\times \frac{v_{n,d}}{\bar{v_{n,d}}}$$ (1),

where

$v_{l}^{s}$: *speed of link l under traffic scenario s*

$v_{l}^{p}$: *speed of link l at time period p*

*v*_*n*,*d*_: *speed in district n on day d*

$$\bar{v_{n,d}}=\frac{\sum_{d}v_{n,d}}{\left|D\right|}$$

s∈S=P×D

*P* = {*p*: *morning*, *afternoon*, *evening*}

*D* = {*d*: *Mon*, *Tue*, *Wed*, *Thr*, *Fri*, *Sat*, *Sun*}

*n*: *local district*

### k-minute coverage calculation

**Fig 3:**
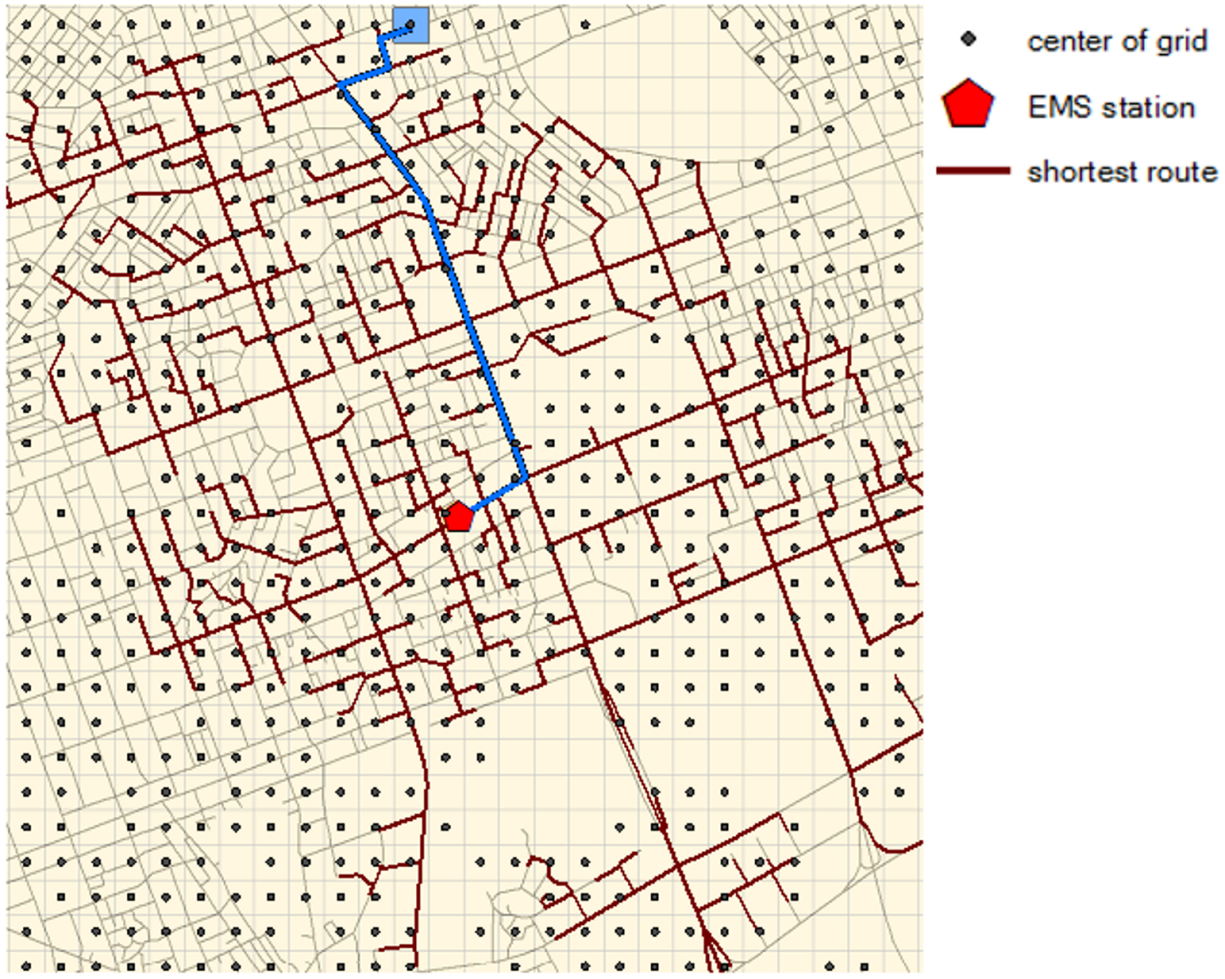
Illustration of per-grid evaluation of k-minute coverage using the shortest-time route.

The study area is partitioned into a 100m by 100m grid, resulting in the total of 29,211 grids. For each grid, the k-minute coverage is calculated based on reachability to the grid from 114 EMS stations within k minutes. The shortest-time travel route on the actual road network is obtained between the closest EMS station to the grid, as illustrated in Fig 3.

Let $t_{g}^{s}$ be the travel time to the center of grid *g* to the closest EMS station under traffic scenario *s*. We define the indicator function 1_*k*_(*g*) of the grid to denote whether the grid *g* is covered within k minutes from one of the EMS stations.

$$1_{k}\left(g\right)=\{\begin{array}{cc} 1, & if\;t_{g}^{s}\leq k\\ 0, & otherwise \end{array}$$ (2)

Now, given a collection of grids *Γ*, the k-minute area coverage ${\text{Area}}_{\Gamma }^{s}\left(k\right)$ and population coverage ${\text{Pop}}_{\Gamma }^{s}\left(k\right)$ is defined as follows.

$${\text{Area}}_{\Gamma }^{s}\left(k\right)=\sum_{\forall g\in \Gamma }1_{k}\left(g\right)$$ (3)

$${\text{Pop}}_{\Gamma }^{s}\left(k\right)=\sum_{\forall g\in \Gamma }1_{k}\left(g\right)\cdot (population\;at\;grid\;g)$$ (4)

Although evaluating 29,211 grids for 21 traffic scenarios might seem cumbersome and computationally expensive, the advantage of such setup is clear once the indicator function value is assigned; one can conduct various analyses using flexible area definition—the whole city, districts or specific area of interest. In this paper, we present the citywide and district-wise analyses.

### Loss in Serviceability due to Traffic (LoST)

To measure the effect of traffic condition to the EMS serviceability, we propose the *Loss in Serviceability due to Traffic (LoST)* defined as follows.

$$LoST_{area}^{s}\left(\Gamma ,k\right)=1-\frac{Area_{\Gamma }^{s}\left(k\right)}{Area_{\Gamma }^{baseline}\left(k\right)}$$ (5)

$$LoST_{area}^{\;}\left(\Gamma ,k\right)=\frac{\sum_{s}LoST_{area}^{s}\left(\Gamma ,k\right)}{\left|S\right|}$$ (6)

$$LoST_{pop}^{s}\left(\Gamma ,k\right)=1-\frac{Pop_{\Gamma }^{s}\left(k\right)}{Pop_{\Gamma }^{baseline}\left(k\right)}$$ (7)

$$LoST_{pop}^{\;}\left(\Gamma ,k\right)=\frac{\sum_{s}LoST_{pop}^{s}\left(\Gamma ,k\right)}{\left|S\right|}$$ (8)

LoST represents the percentage reduction in k-minute coverage compared to the baseline case due to traffic changing conditions. The larger LoST value is, the more severe the effect of traffic in the area of interest is.

To characterize the gaps between the area and population coverage, we calculate the ratio $\rho_{\Gamma }^{k}=\frac{LoST_{pop}^{\;}\left(\Gamma ,k\right)}{LoST_{area}^{\;}\left(\Gamma ,k\right)}$, which naturally yields the elasticity of coverage. In other words, *ρ*_*Γ*_ measures the percent reduction in average population coverage when there is 1% reduction in mean area coverage in *Γ*.

## Results and discussion

### Citywide k-minute coverage

**Fig 4:**
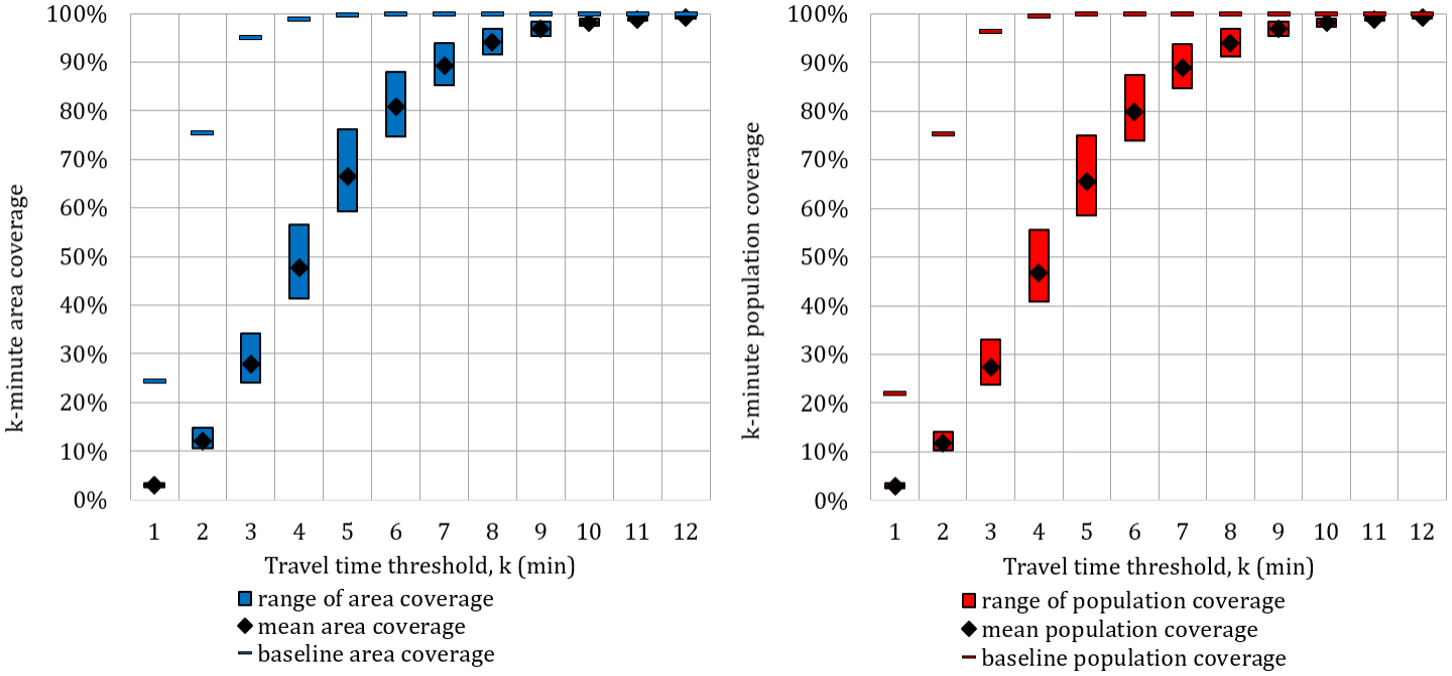
Citywide k-minute coverage distributions. (a) Area coverage. (b) Population coverage.

Distributions of citywide area and population coverage are shown in Fig 4, with travel time threshold k increasing from 1 to 12 minutes. Baseline results of traveling at speed limit show that 75% of city area and population is covered within 2 minutes, and nearly 100% is served within 5 minutes in both categories. When varying traffic scenarios are applied, we find that between 5 and 6 minutes are required to cover 75% of the city on average, and more than 11 minutes are required to serve nearly 100% of city area and population. Considering the 5-minute EMS travel time target, the probability of missing the target is 34.5% for the area and 33.6% for the population on average. In the following section, breakdown analysis of district-wise performance is presented using k = 5 minutes.

### District-wise 5-minute coverage

#### Area coverage

**Fig 5:**
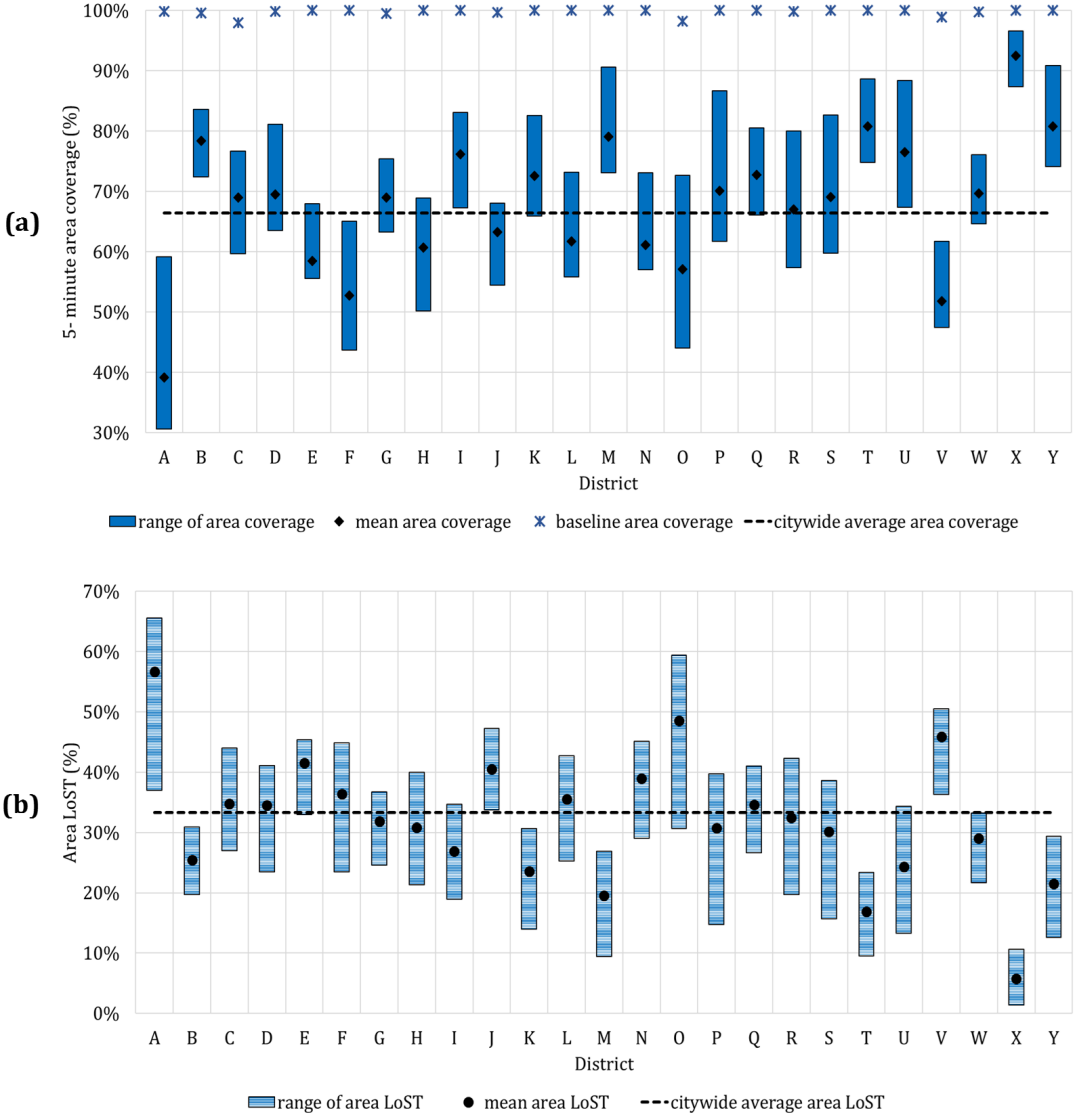
5-minute area coverage by district. (a) Area coverage. (b) Area LoST, $LoST_{area}^{\;s}\left(\Gamma ,5\right)$.

$Area_{\Gamma }^{s}\left(5\right)$ and $LoST_{area}^{\;s}\left(\Gamma ,5\right)$ for all 25 local districts *Γ* ϵ {*A*, *B*, *C*, …, *Y*} along with the baseline case coverage are summarized in Fig 5. We observe that mean coverage area varies greatly by district, ranging from 39.2% to 92.5%. The district-wise *LoST*_*area*_(*Γ*, 5) shows mean and standard deviation of 34.2% and 3.6%. In other words, impact of traffic fluctuation is estimated to reduce the area coverage by 34.2% on average. In the most severe case of district A, coverage reduction is expected to be 56.7% on average and reaches 65.7% in the worst case. One important insight is that coverage reduction is distinctive by district and hardly homogeneous.

#### Population coverage

**Fig 6:**
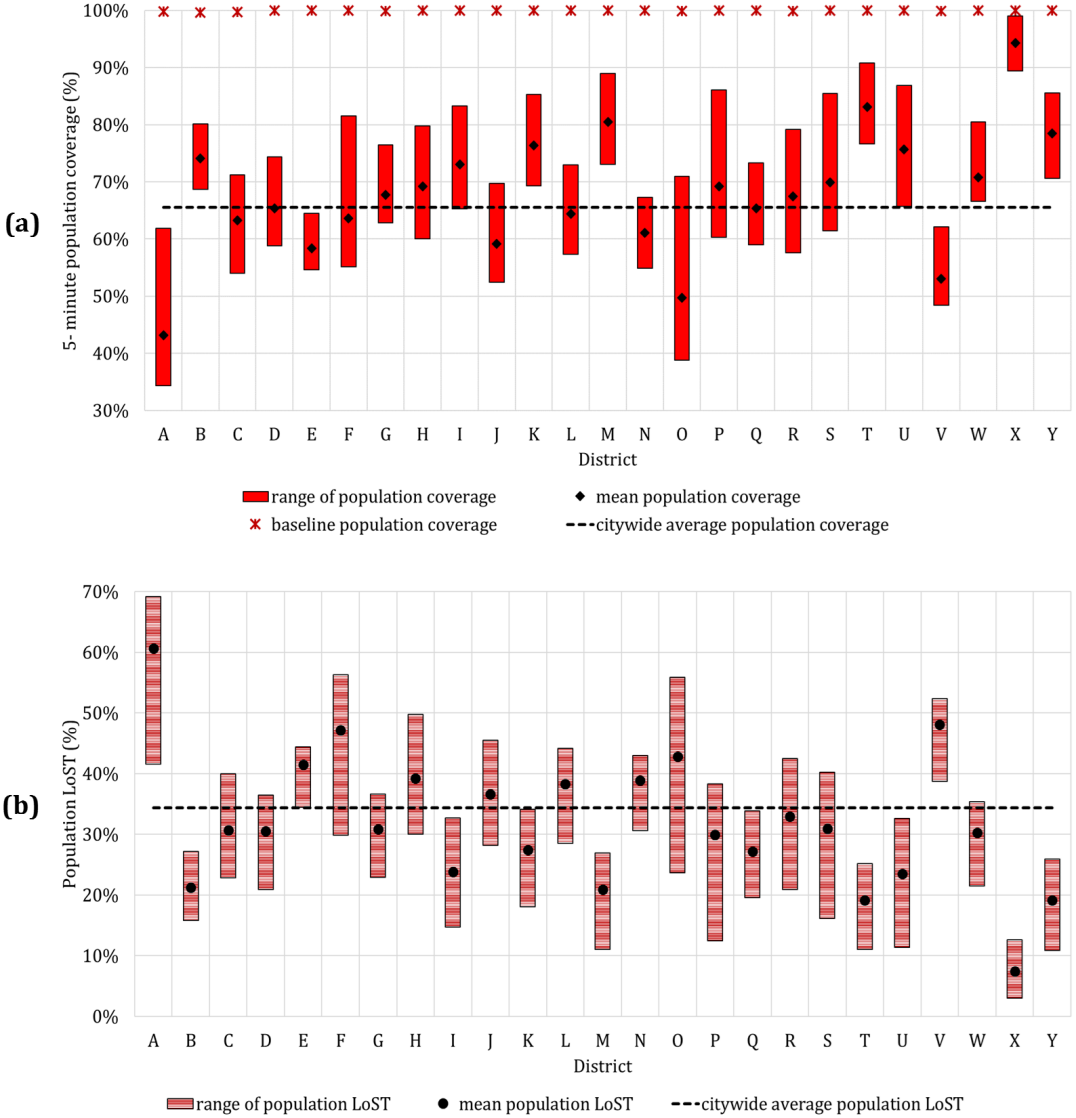
5-minute population coverage by district. (a) Population coverage. (b) Population LoST, $LoST_{pop}^{\;s}\left(\Gamma ,5\right)$.

$Pop_{\Gamma }^{s}\left(5\right)$ and $LoST_{pop}^{\;s}\left(\Gamma ,5\right)$ for all 25 local districts *Γ* ϵ {*A*, *B*, *C*, …, *Y*} along with the baseline case coverage are summarized in Fig 6. We observe a similar trend with the area coverage, with significant variation in 5-minute coverage by district ranging from 43.2% to 94.3%. The *LoST*_*pop*_(*Γ*, 5) shows mean of 33.8% and standard deviation of 3.7%. The district A, which has the most severe reduction in the area coverage turns out to be the top district in population loss with 60.7% reduction on average. Note that some districts such as district B, C, D show larger *LoST*_*pop*_(*Γ*, 5) than *LoST*_*area*_(*Γ*, 5), indicating that area and population reduction is not always proportional.

### Comparison between area and population coverage with ρ_*Γ*_.

**Fig 7:**
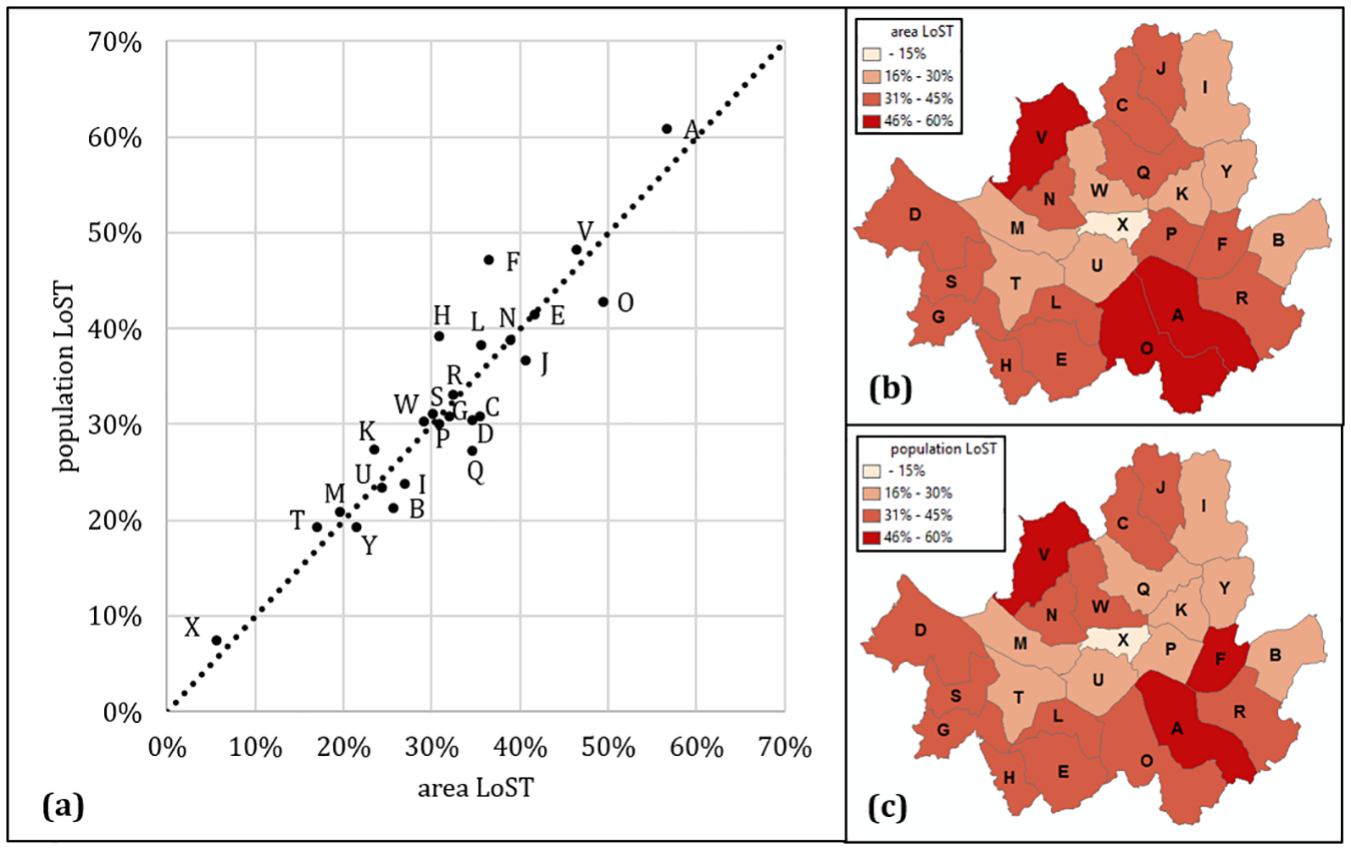
District-wise *LoST*_*pop*_ and *LoST*_*area*_. (a) Scatter plot of mean *LoST*_*pop*_ and *LoST*_*area*_. (b) Geographical illustration of *LoST*_*area*_. (c) Geographical illustration of *LoST*_*pop*_.

In Fig 7, pairwise per-district *LoST*_*pop*_(*Γ*, 5) and *LoST*_*area*_(*Γ*, 5) values are shown in the scatter plot. Overall, the gap between area and population coverage seemed to be well contained. Simple calculation of difference between *LoST*_*pop*_(*Γ*, 5) and *LoST*_*area*_(*Γ*, 5) reveals that 12 of 25 districts show smaller *LoST*_*pop*_ than *LoST*_*area*_, suggesting that half of the city is more sensitive to traffic fluctuation with respect to the serving population than the area. The values of *LoST*_*pop*_ and *LoST*_*area*_ are presented in S1 Table.

**Fig 8:**
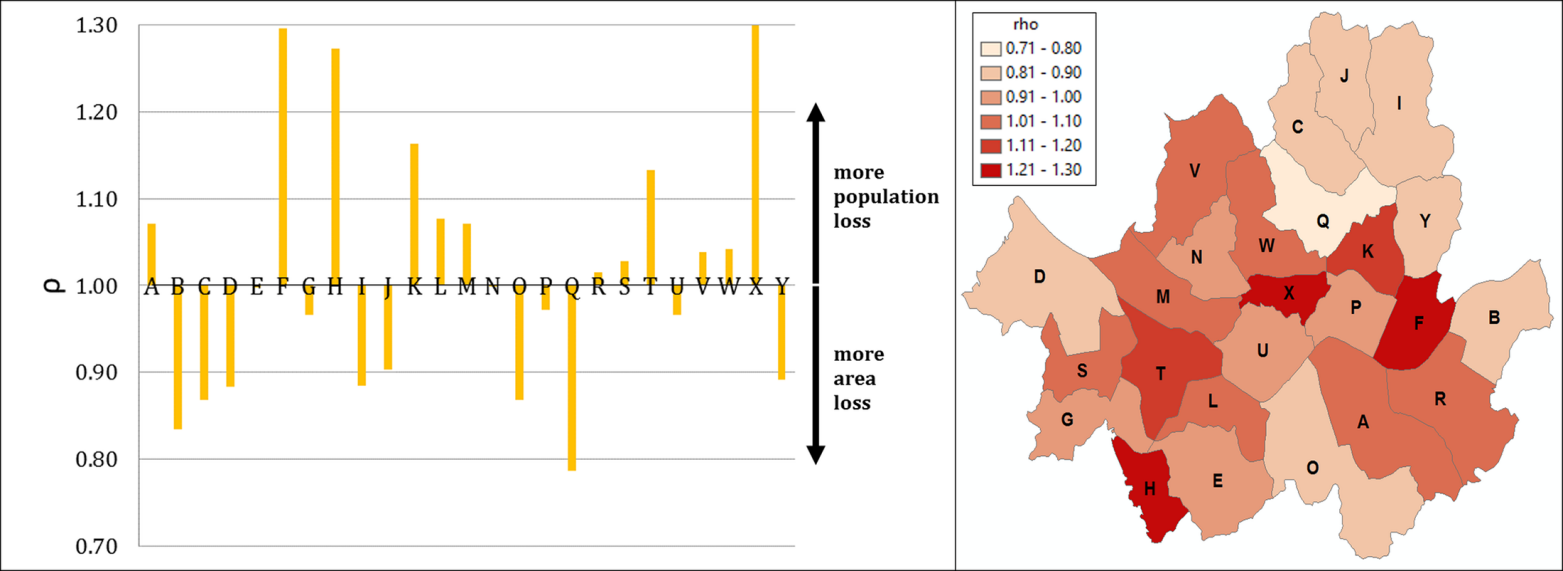
Ratio ρ_*Γ*_ of area and population coverage LoST shown by district.

To further characterize such gaps, the ratio ρ_*Γ*∈{*A*,*B*,*C*,…,*Y*}_ between *LoST*_*pop*_ and *LoST*_*area*_ is calculated and the results are shown in Fig 8. The citywide ρ_*Γ*_ of 0.99 indicates unit elasticity between area and population coverage. However, some districts depart from the citywide unit elasticity. In district Q where the ρ_*Γ*_ has its minimum of 0.78, 1% reduction in area coverage results in 0.78% reduction in population coverage. On the other hand, in district X where the ρ_*Γ*_ has its maximum of 1.31, 1% reduction in area coverage results in 1.31% reduction in population coverage. The wide range of ρ_*Γ*_ suggests that local variations in urban area like Seoul is complex to characterize, and the public health policy such as EMS resource management should take such heterogeneity into consideration and needs to take more adaptive approaches in making the policy decisions and performance evaluation.

## Conclusions

In this paper, we proposed a framework to characterize the influence of traffic fluctuation to EMS first response serviceability in highly urbanized area, and conducted a case study in the city of Seoul, South Korea. Total of 21 traffic scenarios representing the three time-of-day and seven day-of-week variations are generated based on the actual historical traffic data, and imposed on transportation network topology. In addition to adopting the commonly used k-minute coverage concept to evaluate the EMS serviceability, we proposed to include and compare the population coverage as well. The Loss of Serviceability due to Traffic (LoST) is proposed to measure reduction in coverage, and the gap between area and population coverage is analyzed based on the concept of elasticity.

Baseline case of traveling at speed limit shows that almost all city area is served within 5 minutes, which is the performance target enforced by the city. When evaluated for the 21 traffic scenarios against the baseline case, the average citywide reduction in area and population coverage values are similar at 34.2% and 33.8%, respectively. However, district-wise analysis reveals that some districts show more than 50% reduction in both the area and population coverage, while one district shows reductions less than 10% in both categories. We also find that the magnitude of reduction is not always proportional between the area and population coverage, and some districts show nearly 30% more reduction in served population compared to the area coverage.

We conclude that EMS first response performance is highly dependent on the changing condition of transportation infrastructure. Moreover, summarizing such dependency for the city as a whole can be misleading since regional variation is evident among local districts. Although such conclusions are based on our case study, the analysis framework employed in this research can readily be applied to other urban area as long as three data sources are available—speed data, road network topology and population data. Our research demonstrates the importance of understanding the dependency and implication of transportation infrastructure to public health service such as EMS in an urban environment. It is evident that a more adaptive approach is required in urban public health policy decision to plan beyond the simple set of assumptions on related urban infrastructure. We believe that the proposed framework can be effectively incorporated in various EMS policy decisions, including the resource allocation problem as well as the performance target determination such as golden time rule.

## Supporting information

S1 Table Loss in Serviceability due to Traffic (LoST) by district. (PDF)
