# Supplementary material for: Characterizing the influence of transportation infrastructure on Emergency Medical Services (EMS) in urban area—A case study of Seoul, South Korea
Source: PLoS One. 2017 Aug 14;12(8):e0183241. doi: 10.1371/journal.pone.0183241 (PMC5555577; doi:10.1371/journal.pone.0183241)
Supplement: S1 Table — (PDF) [file pone.0183241.s001.pdf]

S1 Table. Loss in Serviceability due to Traffic (LoST) by district

District	$LoST_{area}$				$LoST_{pop}$			
	Mean	Std	CoV	Rank by mean	Mean	Std	CoV	Rank by mean
A	56.7%	7.0%	0.123	1	60.7%	6.7%	0.110	1
B	25.6%	3.3%	0.129	19	21.3%	3.6%	0.167	21
C	35.4%	5.5%	0.155	9	30.8%	5.4%	0.176	13
D	34.5%	4.9%	0.143	11	30.5%	4.4%	0.144	14
E	41.6%	3.2%	0.077	4	41.5%	2.6%	0.063	5
F	36.4%	5.7%	0.158	7	47.2%	7.0%	0.149	3
G	32.0%	3.0%	0.094	13	30.9%	3.6%	0.117	11
H	30.8%	5.8%	0.187	14	39.3%	6.2%	0.158	6
I	26.9%	4.3%	0.159	18	23.8%	4.9%	0.207	19
J	40.6%	4.2%	0.105	5	36.7%	5.5%	0.150	9
K	23.6%	4.7%	0.198	21	27.4%	4.5%	0.162	17
L	35.6%	4.7%	0.133	8	38.3%	4.2%	0.110	8
M	19.5%	4.8%	0.243	23	20.9%	4.1%	0.195	22
N	38.9%	4.1%	0.106	6	38.9%	3.2%	0.083	7
O	49.4%	7.6%	0.154	2	42.9%	8.4%	0.195	4
P	30.8%	6.3%	0.204	14	29.9%	6.3%	0.209	16
Q	34.6%	4.0%	0.116	10	27.2%	4.1%	0.152	18
R	32.5%	6.4%	0.196	12	33.0%	6.1%	0.184	10
S	30.1%	6.1%	0.204	16	30.9%	6.5%	0.211	11
T	16.9%	3.7%	0.222	24	19.2%	3.8%	0.198	23
U	24.3%	5.4%	0.222	20	23.5%	5.3%	0.226	20
V	46.4%	3.7%	0.081	3	48.2%	3.6%	0.074	2
W	29.1%	2.9%	0.101	17	30.3%	3.5%	0.116	15
X	5.7%	3.2%	0.552	25	7.5%	3.1%	0.418	25
Y	21.5%	4.6%	0.215	22	19.2%	4.2%	0.217	23
Citywide	34.2%	3.6%	0.106	-	33.8%	3.7%	0.110	-
